# Supplementary material for: P-TRAP: a Panicle Trait Phenotyping tool
Source: BMC Plant Biol. 2013 Aug 29;13:122. doi: 10.1186/1471-2229-13-122 (PMC3848748; doi:10.1186/1471-2229-13-122)
Supplement: Additional file 7 — P-TRAP source code. The Netbeans project that contains the source code of the application. [file 1471-2229-13-122-S7.zip › javadoc.zip!/javadoc/org-bioalg-mathprocessor/org/bioalg/mathprocessor/BasicMath.html]

BasicMath (MathProcessor)


JavaScript is disabled on your browser.


- Package
- Class
- Tree
- Deprecated
- Index
- Help

*org.bioalg.mathprocessor 1.0*

- Prev Class
- Next Class

- Frames
- No Frames

- All Classes

- Summary:
- Nested |
- Field |
- Constr |
- Method

- Detail:
- Field |
- Constr |
- Method


org.bioalg.mathprocessor

## Class BasicMath

- java.lang.Object
- - org.bioalg.mathprocessor.BasicMath

- ---

    

  ```
  public class BasicMath
  extends java.lang.Object
  ```

  The Basic mathematical operations used in image processing in report
  calculation.

  Author:
  :   faroq

- - ### Constructor Summary

    Constructors

    Constructor and Description

    `BasicMath()`
  - ### Method Summary

    Methods

    | Modifier and Type | Method and Description |
    | --- | --- |
    | `static double` | `angle(java.awt.Point A, java.awt.Point B)` The angel between two points. |
    | `static double` | `eculidian(java.awt.Point A, java.awt.Point B)` The Euclidian distance between two points. |
    | `static double` | `getSlop(java.awt.Point p1, java.awt.Point p2)` Get the slop between two points |
    | `static double` | `getSlopinv(java.awt.Point p1, java.awt.Point p2)` |
    | `static boolean` | `isBoolean(java.lang.String input)` Return true if the input value is boolean. |
    | `static boolean` | `isdouble(java.lang.String input)` Return true if the input value is double |
    | `static boolean` | `isHorizontal(java.awt.Point p1, java.awt.Point p2)` Check if two points are horizontally colinear |
    | `static boolean` | `isInteger(java.lang.String input)` Return true if the input value is integer. |
    | `static boolean` | `IsPerpendicular(java.awt.Point p1, java.awt.Point p2, java.awt.Point p3, java.awt.Point p4)` check if two lines are perpendicular. |
    | `static boolean` | `isVertical(java.awt.Point p1, java.awt.Point p2)` Check if two points are horizontally colinear |
    | `static double` | `max(double[][] matrix)` The maximum of a matrix. |
    | `static double` | `mean(int[] vect)` Return the mean of an integer vector |
    | `static double` | `mean(int[][] mat)` Return the mean of a matrix |
    | `static double` | `pixels2centimeter(double pixels)` Uses the application options to map pixels to centimeters |
    | `static double` | `pixels2Inches(double pixels)` |
    | `static boolean` | `pointInLine(java.awt.Point A, java.awt.Point B, java.awt.Point P)` Return true if a given point is in a line, false otherwise. |
    | `static double` | `pointToLineDistance(java.awt.Point A, java.awt.Point B, java.awt.Point P)` The distance from a point to a line. |
    | `static double` | `roundTO5Decimals(double d)` Round to 5 decimals |
    | `static double` | `roundTO5Decimals2(double d)` |
    | `static double` | `roundTwoDecimals(double d)` Round a double value to two ddecimals |
    | `static double` | `roundTwoDecimals2(double d)` |
    | `static double` | `STD(int[][] mat, double mean)` Return the standard deviation of an integer matrix. |
    | `static double` | `STD(int[] mat, double mean)` Return the standard deviation of vector given its mean |
    | `static double` | `sum(double[] vect)` The sum of double vector. |
    | `static double` | `sum(int[] vect)` The sum of an integer vector |
    | `static double` | `sum(int[][] mat)` The sum of an integer matrix |
    | `static java.awt.Point` | `swapXY(java.awt.Point point)` Swap a point's XY |
    | `static double` | `variance(double[] data)` The variance of a double array. |
    | `static double` | `varinace(double[][] data)` calculate the variance of a matrix |

    - ### Methods inherited from class java.lang.Object

      `clone, equals, finalize, getClass, hashCode, notify, notifyAll, toString, wait, wait, wait`

- - ### Constructor Detail


    - #### BasicMath

      ```
      public BasicMath()
      ```
  - ### Method Detail


    - #### variance

      ```
      public static double variance(double[] data)
      ```

      The variance of a double array.

      Parameters:
      :   `data` - a double array.

      Returns:
      :   a double-valued value.


    - #### varinace

      ```
      public static double varinace(double[][] data)
      ```

      calculate the variance of a matrix

      Parameters:
      :   `data` - the matrix

      Returns:
      :   the variance


    - #### IsPerpendicular

      ```
      public static boolean IsPerpendicular(java.awt.Point p1,
                            java.awt.Point p2,
                            java.awt.Point p3,
                            java.awt.Point p4)
      ```

      check if two lines are perpendicular.

      Parameters:
      :   `p1` - the first point of the first line
      :   `p2` - the second point of the first line
      :   `p3` - the first point of the second line
      :   `p4` - the second point of the second line

      Returns:
      :   true if is perpendicular, false otherwise.


    - #### getSlop

      ```
      public static double getSlop(java.awt.Point p1,
                   java.awt.Point p2)
      ```

      Get the slop between two points

      Parameters:
      :   `p1` - the first point
      :   `p2` - the second point

      Returns:


    - #### isHorizontal

      ```
      public static boolean isHorizontal(java.awt.Point p1,
                         java.awt.Point p2)
      ```

      Check if two points are horizontally colinear

      Parameters:
      :   `p1` - the first point
      :   `p2` - the second point

      Returns:


    - #### isVertical

      ```
      public static boolean isVertical(java.awt.Point p1,
                       java.awt.Point p2)
      ```

      Check if two points are horizontally colinear

      Parameters:
      :   `p1` - the first point
      :   `p2` - the second point

      Returns:


    - #### getSlopinv

      ```
      public static double getSlopinv(java.awt.Point p1,
                      java.awt.Point p2)
      ```


    - #### roundTwoDecimals

      ```
      public static double roundTwoDecimals(double d)
      ```

      Round a double value to two ddecimals

      Parameters:
      :   `d` - the input double

      Returns:


    - #### roundTwoDecimals2

      ```
      public static double roundTwoDecimals2(double d)
      ```


    - #### roundTO5Decimals

      ```
      public static double roundTO5Decimals(double d)
      ```

      Round to 5 decimals

      Parameters:
      :   `d` - the input double.

      Returns:


    - #### roundTO5Decimals2

      ```
      public static double roundTO5Decimals2(double d)
      ```


    - #### pixels2centimeter

      ```
      public static double pixels2centimeter(double pixels)
      ```

      Uses the application options to map pixels to centimeters

      Parameters:
      :   `pixels` - a double value represent the number of pixels

      Returns:
      :   a double value represents the centimeters


    - #### pixels2Inches

      ```
      public static double pixels2Inches(double pixels)
      ```


    - #### sum

      ```
      public static double sum(int[] vect)
      ```

      The sum of an integer vector

      Parameters:
      :   `vect` - an integer vector.

      Returns:
      :   a double value of the sum


    - #### sum

      ```
      public static double sum(double[] vect)
      ```

      The sum of double vector.

      Parameters:
      :   `vect` - the vector

      Returns:
      :   the sum of the vector.


    - #### sum

      ```
      public static double sum(int[][] mat)
      ```

      The sum of an integer matrix

      Parameters:
      :   `mat` - the integer matrix

      Returns:
      :   the sum value.


    - #### mean

      ```
      public static double mean(int[] vect)
      ```

      Return the mean of an integer vector

      Parameters:
      :   `vect` - the integer vector

      Returns:
      :   the sum


    - #### mean

      ```
      public static double mean(int[][] mat)
      ```

      Return the mean of a matrix

      Parameters:
      :   `mat` - the matrix

      Returns:
      :   the mean value.


    - #### STD

      ```
      public static double STD(int[] mat,
               double mean)
      ```

      Return the standard deviation of vector given its mean

      Parameters:
      :   `mat` - the vector
      :   `mean` - the mean

      Returns:
      :   the std.


    - #### STD

      ```
      public static double STD(int[][] mat,
               double mean)
      ```

      Return the standard deviation of an integer matrix.

      Parameters:
      :   `mat` - the matrix
      :   `mean` - the mean

      Returns:
      :   the std.


    - #### isInteger

      ```
      public static boolean isInteger(java.lang.String input)
      ```

      Return true if the input value is integer.

      Parameters:
      :   `input` -

      Returns:


    - #### isdouble

      ```
      public static boolean isdouble(java.lang.String input)
      ```

      Return true if the input value is double

      Parameters:
      :   `input` -

      Returns:


    - #### isBoolean

      ```
      public static boolean isBoolean(java.lang.String input)
      ```

      Return true if the input value is boolean.

      Parameters:
      :   `input` -

      Returns:


    - #### swapXY

      ```
      public static java.awt.Point swapXY(java.awt.Point point)
      ```

      Swap a point's XY

      Parameters:
      :   `point` -

      Returns:


    - #### pointToLineDistance

      ```
      public static double pointToLineDistance(java.awt.Point A,
                               java.awt.Point B,
                               java.awt.Point P)
      ```

      The distance from a point to a line.

      Parameters:
      :   `A` - The first pint in the line
      :   `B` - The second point in the line.
      :   `P` - The point.

      Returns:
      :   a double value.


    - #### pointInLine

      ```
      public static boolean pointInLine(java.awt.Point A,
                        java.awt.Point B,
                        java.awt.Point P)
      ```

      Return true if a given point is in a line, false otherwise.

      Parameters:
      :   `A` - The first point in the line
      :   `B` - The last point in the line.
      :   `P` - The point.


    - #### eculidian

      ```
      public static double eculidian(java.awt.Point A,
                     java.awt.Point B)
      ```

      The Euclidian distance between two points.

      Parameters:
      :   `A` - The first point
      :   `B` - The second point

      Returns:
      :   a double value.


    - #### angle

      ```
      public static double angle(java.awt.Point A,
                 java.awt.Point B)
      ```

      The angel between two points.

      Parameters:
      :   `A` - the first point
      :   `B` - the second point


    - #### max

      ```
      public static double max(double[][] matrix)
      ```

      The maximum of a matrix.

      Parameters:
      :   `matrix` -

      Returns:


- Package
- Class
- Tree
- Deprecated
- Index
- Help

*org.bioalg.mathprocessor 1.0*

- Prev Class
- Next Class

- Frames
- No Frames

- All Classes

- Summary:
- Nested |
- Field |
- Constr |
- Method

- Detail:
- Field |
- Constr |
- Method
